# Nanoformulation of Paclitaxel: Exploring the Cyclodextrin / PLGA Nano Delivery Carrier to Slow Down Paclitaxel Release, Enhance Accumulation *in Vivo*

**DOI:** 10.7150/jca.82410

**Published:** 2023-03-27

**Authors:** Peilin Ma, JiaYing Huang, Jinling Liu, Yi Zhu, Jiahong Chen, Junming Chen, Lunwen Lei, Ziyun Guan, Junfeng Ban, Zhufen Lu

**Affiliations:** 1 Guangdong Pharmaceutical University, Guangzhou, People's Republic of China; 2 The Sixth Affiliated Hospital, School of Medicine, South China University of Technology, Guangzhou, People's Republic of China; 3 The Innovation Team for Integrating Pharmacy with Entrepreneurship, Guangdong Pharmaceutical University, Guangzhou, People's Republic of China; 4 Guangdong Provincial Key Laboratory of Advanced Drug Delivery Systems, Guangdong Pharmaceutical University, Guangzhou, People's Republic of China; 5 Guangdong Provincial Engineering Center of Topical Precision Drug Delivery System, Center for Drug Research and Development, Guangdong Pharmaceutical University, Guangzhou, People's Republic of China

**Keywords:** PLGA nanoparticles, 2-HP-β-CD, *in vivo* imaging, pharmacokinetics.

## Abstract

**Background:** Improving the aggregation and penetration in tumor sites increases the anti-tumor efficacy of nanomedicine. In the current study, we designed cyclodextrin modified PLGA nanoparticles loaded with paclitaxel to elevate the accumulation and prolong circulation of chemotherapy drugs *in vivo*.

**Methods:** The PLGA nanoparticles loaded with paclitaxel (PTX PLGA NPs) and cyclodextrin (CD) modified PLGA nanoparticles loaded with paclitaxel (PTX PLGA/CD NPs) were prepared using the emulsification solvent evaporation method. The nanoparticles were characterized by particle size, zeta potential, encapsulation efficiency, infrared spectroscopy analysis and X-Ray diffraction (XRD). Then, drug release of the nanoparticles was evaluated via reverse dialysis method *in vitro*. Finally, the *in vivo* distribution fate and pharmacokinetic characteristics of the nanoparticles were assessed in mice and rats.

**Results:** The average particle size, zeta potential, and encapsulation efficiency of PTX PLGA NPs were (163.57±2.07) nm, - (20.53±2.79) mV and (60.44±6.80)%. The average particle size, zeta potential, and encapsulation efficiency of PTX PLGA/CD NPs were (148.57±1.66) nm, - (11.42±0.84) mV and (85.70±2.06)%. *In vitro* release studies showed that PTX PLGA/CD NPs were released more slowly compared to PTX PLGA NPs under normal blood pH conditions, while PTX PLGA/CD NPs were released more completely under tumor site pH conditions. The modified PLGA nanocarrier (PLGA/CD NPs) increased drug residence time and accumulation than the plain PLGA nanocarrier (PLGA NPs) *in vivo* distribution. In addition, the elimination half-life, area under the drug-time curve, and maximum blood concentration of the nanoparticle group were higher than those of Taxol^®^, especially the PTX PLGA/CD NPs group, which was significantly different from Taxol^®^ and plain nanoparticle groups (*p*<0.001).

**Conclusions:** The 2-HP-β-CD modified PLGA nanoparticles prolonged circulation time and accumulation of the chemotherapy drug paclitaxel *in vivo*.

## Introduction

Paclitaxel, as a first-class broad-spectrum chemotherapy drugs, was widely applied in clinical practice, with good efficacy in a variety of solid tumors, including triple-negative breast cancer, non-small cell lung cancer, and ovarian cancer 1-3. However, paclitaxel has poor water solubility and tissue selectivity. The solubility of paclitaxel was measured in PEG 400, ethanol, octanoic acid, oleic acid and miglidol 812 using the shake flask method. The solubility of paclitaxel in PEG 400 is greater than 125mg·mL^-1^ because the drug may interact hydrophobically with polyethylene chains. The solubility of paclitaxel in ethanol is greater than 52mg·mL^-1^, and the solubility of paclitaxel in octanoic acid, oleic acid and miglidol 812 is also higher than its water solubility, which is 2.22mg·mL^-1^, 0.55mg·mL^-1^ and 1.05mg·mL^-1^, respectively 4. The issues associated with conventional chemotherapy have prompted the development of a wide-range of nanocarriers for safer and more effective delivery of chemotherapeutics. These include inorganic (e.g., silica 5 and silver 6) and organic (e.g., polymers 7 and cellular membranes 8) nanoparticles (NPs). Among these NPs, PLGA NPs, which are composed of a stealth hydrophilic shell surrounding a hydrophobic core encapsulating a variety of water insoluble drugs, have garnered considerable interest 9. Some researchers have confirmed the safety of intravenous administration of paclitaxel-loaded PLGA nanoparticles and that PLGA allows good efficacy and slow release of paclitaxel *in vivo* 10-13. PLGA nanoparticles can be coated with modification materials on the surface to achieve passive, active, and physical targeting pathways, thus enriching to tumors for deeper drug delivery.

Various studies have incorporated nanoparticle design's surface charge, size, and mechanical properties to enhance the tumor site permeability. Wang reported that Polyoxybutylene-polyoxyethylene encapsulated nanoparticles reduce phagocytosis of the reticuloendothelial system and improve tumor aggregation 14. The fluorescence intensity of nanoparticles in the tumor was twice that in the liver after six hours of intravenous injection. In contrast, the fluorescence intensity of PLA-PEG nanoparticles with similar size in the tumor was identical to that in the liver, indicating that the PBO-PEG nanoparticles could reduce liver and spleen uptake and improve tumor aggregation. Reportedly, nano-drugs can overcome the tumor biological barrier and achieve deep drug delivery through responsive adjustments or transformations such as hydration layer shedding, charge inversion, variable particle size, multi-drug loading, and sequential release in different biological environments 15, 16. Zhou et al. found that tiny drug particles tend to shuttle within the intercellular space. In contrast, large particles are more likely to travel between cells. According to the specific demand to control the size and charge, an appropriate drug penetration mode can be selected to improve nano-drug tumor permeability 17. Yet, to date, very few of these NPs systems have reached the clinical trial stage 18, 19. One of the few successes of this strategy so far is mPEG-b-PDLLA loaded with paclitaxel (Genexol-PM), which is currently approved for the treatment of metastatic breast cancer, non-small cell lung cancer and ovarian cancer. Although they have been shown to reduce some of the adverse effects associated with chemotherapeutics, block copolymer micelles do not yield the expected enhancement in therapeutic outcome relative to free drugs 20. In order to improve the anti-tumor efficacy of nano-drugs, existing studies often combine a variety of methods to achieve synergy, but too complex design and tedious preparation process will hinder the practicability of research results and the feasibility of clinical transformation. To address these challenges, we have designed hybrid NPs that consist of a polylactic-co-glycolic acid (PLGA) core 'wrapped' with a hydrophilic cyclodextrin shell that is functionalized with the tumor site pH conditions. In this study, 2HP-β-CD hydrophilic cyclodextrin was used to modify the surface of PLGA nanoparticles, and its properties were evaluated in order to explore the outstanding problem of poor permeability of nano-drugs in tumors.

## Materials and Methods

### Materials and animals

The drug paclitaxel(PTX) was purchased from Hainan Yew Park Pharmaceutical Co., Ltd. (Hainan, People's Republic of China); Taxol® from LaiMei Pharmaceutical Co., Ltd. (Chongqing, People's Republic of China); PLGA (LA: GA = 75/25, MW=10k) from Dai Gang Bioengineering Co., Ltd. (Jinan, People's Republic of China); Polyvinyl alcohol (PVA) was provided by Kuraray International Trading Co., Ltd. (Shanghai, China); 2-hydroxypropyl-β-cyclodextrin(2-HP-β-CD,CD) from Qian Hui Biotechnology Co., Ltd. (Zibo, People's Republic of China). Rhodamine-B from DaMao Chemical Reagent Co., Ltd. (Tianjin, People's Republic of China); All the other reagents used were of the highest commercial grade.

We purchased KM Mice (female and male, weighing 18-22g) and SPF grade rats (female and male, weighing 180-220g) from the Experimental Animal Center of Guangzhou University of Traditional Chinese Medicine. The study was approved by the Institutional Animal Care and Use Committee of Guangdong Pharmaceutical University. The committee ensured that animal usage and care complied with the National Institutes of Health guidelines.

### Preparation of the PTX PLGA NPs and PTX PLGA/CD NPs

The emulsification solvent evaporation method was adopted to prepare paclitaxel-loaded PLGA nanoparticles (PTX PLGA NPs) and paclitaxel-loaded PLGA/CD nanoparticles (PTX PLGA/CD NPs) 21-23. Paclitaxel and PLGA (PTX: PLGA, 1mg: 30mg) were weighed and dissolved in 3mL measured volume of acetone: ethanol (8:2) mixture as the oil phase. 2% PVA aqueous solution or 2% 2-HP-β-CD aqueous solution was the water phase. Under probe sonication, the oil phase was injected into the water phase for 10 minutes of ultrasonic emulsification (the horn is 6mm, the power is 150kHz). Then, the oil solvent was removed by magnetic stirring for 8h.

To depict the ability of the nanocarrier to encapsulate insoluble drug,we first separated the nanoparticles from the free PTX with a Sephadex G-50 column (1.5cm×15cm) chromatography. Loading volume: 0.5 mL; Collection volume per tube: 2 mL; Elution medium: 0-30mL deionized water and 31-60mL 5% sodium dodecyl sulfate (SDS) aqueous solution.

### Characterization of the PTX PLGA NPs and PTX PLGA/CD NPs

#### Size and Zeta Potential

Particle size and surface electric potential are crucial indicators for nanoparticles that closely related to the *in vivo* behavior of nanoparticles. The Delsa nano C laser particle sizer (Beckman Coulter Inc., California, USA) was introduced. The particle size, D90 (the value of the particle size distribution corresponding to 90%), polydispersity index (PDI) and distribution were determined by Dynamic light scattering (DLS), and the zeta potential was determined by Electrophoretic light scattering (ELS), and the dispersion medium was deionized water. The particle size was expressed as the average intensity diameter (±SD of value).

#### Surface Morphology

Taken the PTX PLGA NPs and PTX PLGA/CD NPs solution and dropped onto the copper mesh covered with carbon film, stained with 2% phosphotungstic acid, and dried naturally. The surface morphology of the nanoparticles was observed under transmission electron microscopy (TEM) at 50,000 times magnification.

#### Encapsulation efficiency

Add 3mL of methanol to the purified nanoparticles to break the emulsion. The encapsulation efficiency (EE) of the purified nanoparticles was measured using high-performance liquid chromatography (HPLC) and calculated with the following formula 24:


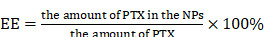

(1)

Waters (e2695 HPLC system, USA) HPLC equipped with a Kromasil C18 column (4.6×250mm, 5μm) was used to determine EE. The mobile phase included methanol: water (65:35, v/v). The injection volume was 20μL, and the flow rate was 1mL·min^-1^. The column temperature was 30 °C, and the detection wavelength was 227nm.

### Infrared spectroscopy analysis (IR)

Freeze-drying technology is used to freeze-dry the nanoparticles, the pre-freezing procedure is -20℃ for 4h and then transferred to -80℃ for 8h, and finally transferred to freeze-dryer (Free zone 6L, LABCONCO Inc) for 24h.

The position and intensity of infrared absorption peaks in infrared spectroscopy reflect structural molecular characteristics. Therefore, they can identify the structural composition of unknown molecules and determine their chemical groups.

Thus, infrared spectroscopy (Spectrum 100, PerkinElmer) measured the characteristic absorption spectra of the nanoparticles, PTX, and physical mixture (excipients and paclitaxel, PM).

3mg of the freeze-dry the nanoparticles sample was mixed with 100mg dry KBr powder, grounded, and pressed with the tablet, and the scanning wavelength was set at 400-4000cm^-1^.

#### X-ray diffraction (XRD)

X-ray diffraction (D8-Advance, Bruker Co., Germany) analysed the crystal state of the drug within the nanoparticles. X-ray diffraction analyzed paclitaxel, PM and nanoparticles. The test conditions included: copper target, tube pressure: 40Kv, tube current: 40mA, scanning 5-45° at a 2θ angle.

### Drug release of the PTX PLGA NPs and PTX PLGA/CD NPs

The pH of blood, the tumor site, and the lysosome in tumor cells are about 7.4, 6.8, and 5.0, respectively 25. To reflect the fate of nanoparticles circulating *in vivo*, we simulated different pH environments. The reverse dialysis method was applied to investigate the PTX release properties of PTX-PLGA NPs and PTX-PLGA/CD NPs. The phosphate buffer solutions at different pH were used as the release medium, and the solubilizer was 1% sodium dodecyl sulfate (SDS). The nanoparticles were added in a dialysis bag with 8000-14000 molecular weight cut-off and dipped into a beaker flask containing 30 mL PBS (pH = 7.4, 6.8, 5.0). Then, it was put in a shaker (120 rpm, 37 °C). Around 5mL of the release medium was taken out at a specific time point (0.5h, 2h, 4h, 6h, 8h, 12h, 24h, 48h, 72h, 96h, 120h, 240h, 360h, 720h) and replaced with 5mL fresh release medium. The paclitaxel concentration was determined and calculated by HPLC. The cumulative drug release rate was calculated with the following formula 4. Each experiment was repeated in triplicates.


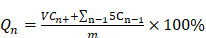

(2)

Q: cumulative drug release rate, %; *V*: total volume of release medium, 30mL; C_n:_ concentration of drug in the release medium at the nth sampling, μg·mL^-1^; m: total amount of drug encapsulated by nanoparticles, μg; n: number of sampling.

### Pharmacokinetics

All animal protocols complied with the Guide for the Care and Use of Laboratory Animals and Institute of Laboratory Animal Resources and were approved by the Institutional Animal Care and Use Committee of Guangzhou Quality Supervision and Testing Institute (2021-08-03). Healthy SD rats, weighing 180-220g, were randomly divided into two groups (6 pcs per group) to know the pharmacokinetic properties of nanoparticles in the *in vivo* environment 26, 27. Commercial paclitaxel and purified PTX PLGA/CD NPs were injected into KM mice at a 20mg·kg^-1^ dose through the tail vein. After 5min, 15min, 30 min, 1, 2, 3, 4, 8 and 12 h post-injection, 0.25mL blood was taken from orbit and placed in a heparinized centrifuge tube. The upper plasma layer was taken after centrifuging the blood sample at 6000rpm for 10 minutes. The obtained plasma samples were processed as follows: 100 μL of plasma samples were vortexed, sonicated for 1 minute, and then extracted in 200 μL of acetic acid. After centrifugation at 6000rpm for 30 minutes, the supernatant was collected and evaporated using nitrogen. The residue was replaced with 100 μL of methanol and analyzed using HPLC. The DAS 3.0 software helped in calculating the pharmacokinetic parameters. The content of paclitaxel in plasma was determined by HPLC. On the basis of reference to the determination of preparation, the mobile phase ratio was adjusted to methanol-water ratio at 70:30 to avoid the interference of endogenous substances.

### Body accumulation

The distribution of nanoparticles in mice was investigated using near-infrared *in vivo* imaging to evaluate the targeting of nanoparticles *in vivo* better 28. NPs loaded with rhodamine-B (RDM) were prepared by replacing PTX with RDM. Then, physiological saline (negative control group), RDM PLGA NPs and RDM PLGA/CD NPs were injected into KM mice at a 15mg·kg^-1^ dose through the tail vein. After 5 minutes, 30 minutes, 2 hours, 4 hours, and 6 hours post-injection, the *in vitro* imaging signal of RDM in major organs were monitored. The mice were sacrificed after six hours of the administration, and the major organs (heart, liver, spleen, lung, kidney, and brain) were removed. The tissue was washed three times with saline and placed in a watch glass for better imaging. For imaging, the excitation wavelength was 550nm, the emission wavelength was 600nm, and the exposure time was fixed to 0.1s.

### Statistical analysis

Data were expressed as the mean ± standard deviation (SD) of three independent experiments. An analysis of variance assessed differences amongst the groups. Intragroup group differences were evaluated using an independent sample t-test, using SPSS software (SAS Institute, Cary, NC, USA). *p<0.05* was considered statistically significant.

## Results and Discussion

### Characterization of the PTX PLGA NPs and PTX PLGA/CD NPs

**Figure 1:**
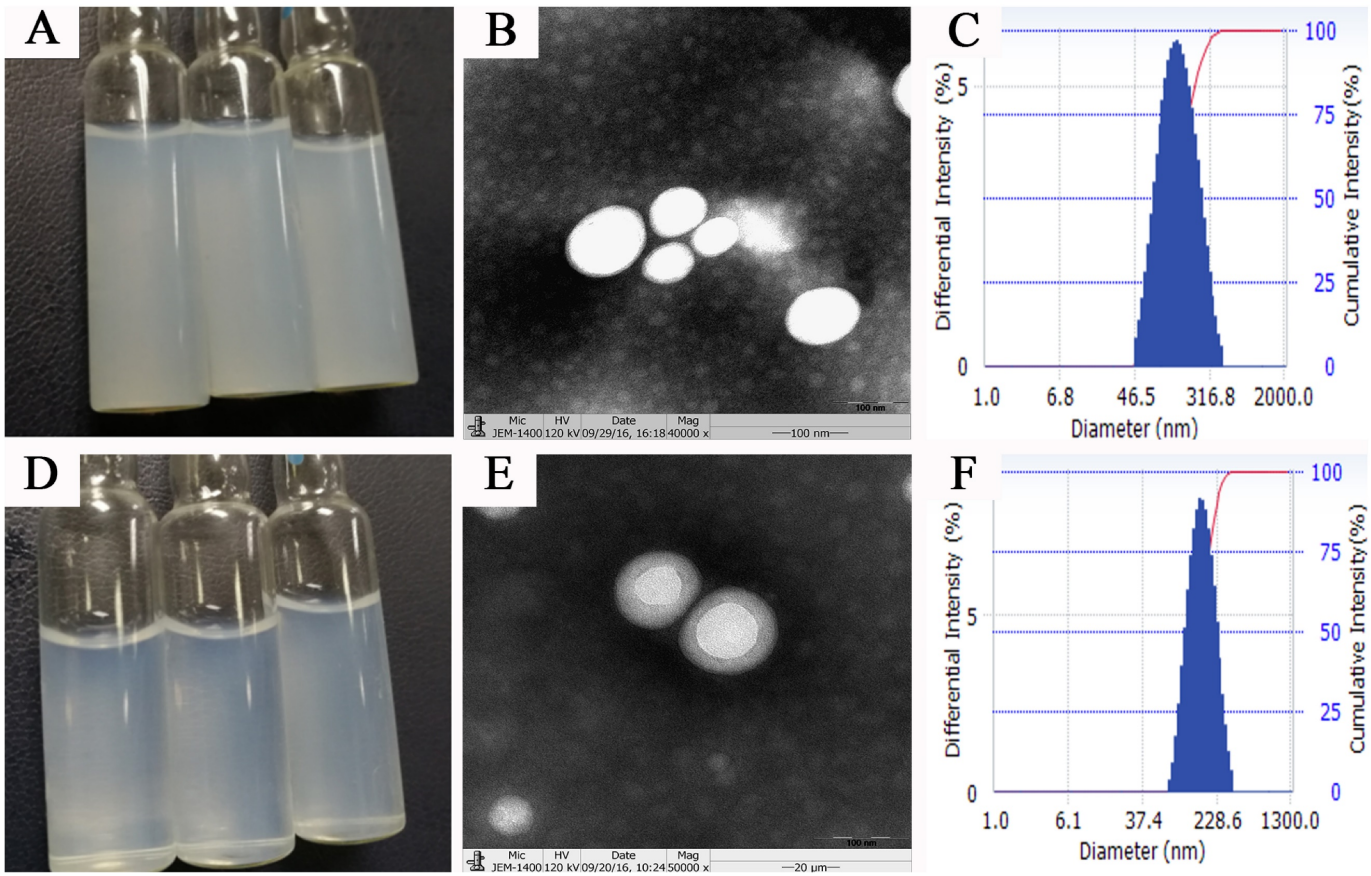
Nanoparticles appearance, micromorphology and size distribution (A,B,C: appearance、TEM×50000 figure and size distribution of PTX PLGA NPs; d,e,f: appearance, TEM×50000 figure and size distribution of PTX PLGA/CD NPs)

Surface properties such as particle size, surface electric potential, and morphology of nanoparticles are crucial quality indicators and closely related to the *in vivo* fate of nanoparticles. PTX PLGA NPs and PTX PLGA/CD NPs were prepared by emulsification solvent evaporation method, and the microscopic morphology, average particle size and size distribution, surface electric charge, and encapsulation rate were employed as evaluation indexes. The aqueous solution of PTX PLGA NPs showed a translucent light blue opalescent solution and the aqueous solution of PTX PLGA/CD NPs showed a clarified and transparent light blue opalescent solution (Figure 1A, 1D). Both nanoparticles were found to be spherical in shape under transmission electron microscopy, and the CD-modified nanoparticles had a more regular morphology and were surrounded by the CD adsorbed on the PLGA surface (Figure 1B, 1E).

The average particle size and ζ potential of nanoparticles before and after CD modification were determined, and the results are shown in Table 1. The average particle size of nanoparticles before and after CD modification were (163.57±2.07) nm and (148.57±1.66) nm, respectively, and the average particle size after CD modification was significantly smaller than that of plain nanoparticles. According to the EPR effect, nanoparticles of 100nm-200nm size can better accumulate in tumor sites 16. And from the particle size distribution diagram (Figure 1C, F), we can see that the particle size distribution range decreases after CD modified, and the D90 value decreases. This result suggests better stability of the PTX PLGA/CD NPs as zeta potential with a higher negative magnitude value representing the nanoparticle's stability. It can be attributed to the presence of CD that is associated with improved stability of nanoparticles 29.

Meanwhile, the inner surface cavity of CD could encapsulate part of the paclitaxel drug, so the encapsulation rate increased after CD encapsulation (Table 1). The ζ potentials before and after modification were -(20.53±2.79) mV and -(11.42±0.84) mV, respectively, and the surface electronegativity of nanoparticles was significantly reduced after modification.

From the PDI results, it is clear that the CD/PLGA nanoparticles can significantly improve the system stability with a PDI at 0.14 compared to 0.28 for the plain PLGA nanoparticles.

### IR and XRD

**Figure 2:**
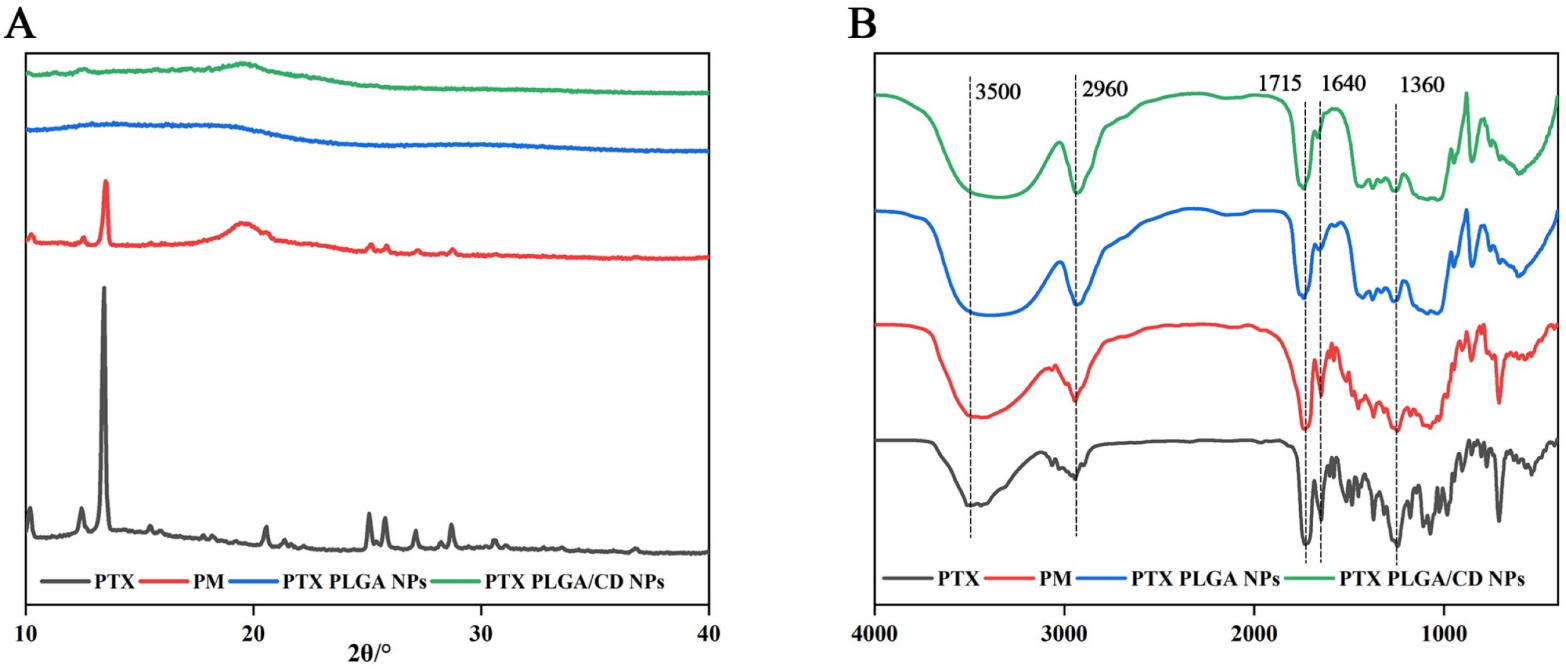
(A) X-ray diffraction patterns of NPs; (B) Infrared spectrograms of NPs.

The poly (lactic acid-glycolic acid) bearing hydroxyl end group diblock copolymer as a nanoparticles carrier to prolong their circulation time when administered in the body and reduce their intermolecular aggregation through inter-particle steric repulsion 30. X-ray diffraction can be used to determine the presence state of paclitaxel in different nanoparticles. From Figure 2A, it can be seen that paclitaxel has a strong diffraction peak between 10-20(2θ/°), and in the diffraction map of physical mixture, the characteristic peak at the corresponding position appears, but it is relatively weak, and the diffraction line widens in the high angle, probably because the microcrystalline grains become smaller in size after grinding, and because there are many amorphous substances such as PVA and PLGA caused the specimen to be poorly crystallized, so the diffraction lines were broadened, but it can still indicate that the drug was dispersed in the mixture in crystalline form, and the crystalline shape did not change in the physical mixture. In contrast, in PLGA nanoparticles and CD/PLGA nanoparticles, the characteristic diffraction of paclitaxel disappeared, and the crystallinity of paclitaxel was reduced in the nanoparticles, and paclitaxel might be completely dissolved or existed in the nanoparticles in an amorphous state, and this state was favorable to improve the stability of the drug.

FTIR spectral analysis of PTX PLGA NPs, and PTX PLGA/CD NPs vibrational modes in their native form and nanoparticle formulations provides insight into the structural integrity and retention of functional groups in the nanoparticles formed 31. Figure 2B shows the physical mixture of paclitaxel, paclitaxel and carrier material (physical mixture, PM. Made by mixing 1mg paclitaxel and 30 mg PLGA homogeneously), PTX PLGA NPs, and PTX PLGA/CD NPs. The main characteristic absorption peaks of paclitaxel were 3500cm^-1^(-OH vibration peak), 2960cm^-1^(=C-H hydrocarbon bond), 1715cm^-1^ (C=O carbonyl ketone group stretching vibration), 1640cm^-1^(CONH_2_ amide group stretching vibration), 1360cm^-1^(CH_3_ methyl bending vibration); the same characteristic absorption peaks were present in the physical mixture. indicating that no significant chemical interaction occurred between paclitaxel and the carrier, and the same characteristic absorption peaks could be observed in the two nanoparticle spectra, indicating that no significant chemical interaction occurred between paclitaxel and the carrier material.

### Drug release of the and PTX PLGA/CD NPs

**Figure 3:**
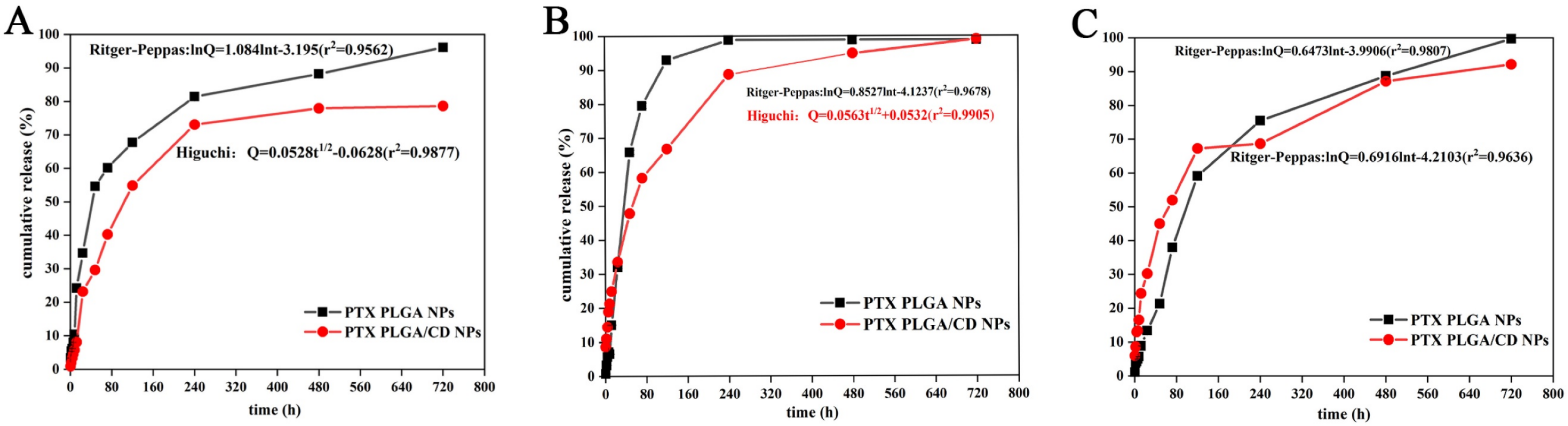
The cumulative release rate of NPs in other pH conditions. (A: in pH 5.0 buffer; B: in pH 6.8 buffer; C: in pH 7.4 buffer).

The *in vitro* release behavior of nanodrugs can reflect the *in vivo* release of nanodrugs to some extent. To demonstrate the pH-sensitivity of CD based PLGA, PTX release from PLGA NPs, and PLGA/CD NPs samples were investigated at three different pH conditions (pH 5.0, 6.8 and 7.4) using dialysis method (Figure 3) 25. Limited PTX release was noticed at pH 7.4 for all samples. Specifically, after 3h of incubation, the slow release at pH 7.4 can be interpreted based on the hydrophobic PTX PLGA NPs at a relatively high pH, which increased the hydrophobic interaction between PTX and the PLGA core. Moreover, for PLGA/CD NPs samples, the existence of intact crosslinked core/shell intermediate layer retarded diffusion of ptX from NPs core. Previous work by Mohamed et al. ascribes a limited release of DOX at pH 7.4 for the same reason 32.

To simulate the *in vivo* physiological conditions, pH 7.4(pH of normal blood environment), pH 6.8 (pH of tumor site) and pH 5.0 (pH inside tumor cells) were taken. From the three release profiles, it can be found that the cumulative release rate of two kinds of nanoparticles reached more than 80% in both groups under each pH condition. The release of paclitaxel was delayed by CD-modified nanoparticles compared to PLGA nanoparticles under all pH conditions, especially under normal blood pH conditions. The release rate of CD-modified nanoparticles under tumor tissue and tumor cell pH conditions was significantly greater than that of the CD-modified nanoparticle group under normal blood pH conditions, and the release was complete under tumor site pH conditions, with a cumulative release rate of more than 90%. The cumulative release rate of the nanoparticle group under tumor site pH conditions reached more than 90% with complete release.

Further, the release behavior of nanoparticles in each group under each pH condition was evaluated by using origin software. The best fit results are shown in Figure 3 insert equation. Under normal blood pH conditions, the release behavior of CD-modified nanoparticle group showed the characteristics of Ritger-Peppas equation and the diffusion coefficient was 0.7057, which was in the range of 0.45-0.85, indicating that the drug release mechanism was a combination of Fick diffusion and Skeleton dissolution. And the CD-modified nanoparticle set fitted better with the Higuchi equation under tumor site and tumor cell pH conditions. The results of release curves and fitted equations indicate that PTX PLGA/CD NPs can be released faster and more completely under tumor micro-acid conditions. This provides favorable conditions for prolonged accumulation of paclitaxel drug at the tumor site.

### Pharmacokinetics

*In vivo* pharmacokinetic experiments in rats can visualize the effect of the formulations circulating 33. DAS3.0 software was used to fit the plasma drug concentrations of different formulations at various time points, and the results showed that the nanoparticle groups (PTX PLGA NPs and PTX PLGA/CD NPs) were consistent with the two-compartment model (Table 2).

The elimination half-life, mean residence time, area under the drug-time curve, and maximum blood concentration of the nanoparticle group were higher than those of Taxol®, especially the CD-modified nanoparticle group, which was significantly different from Taxol® and plain nanoparticle groups (*p<0.001*). The MRT of PTX PLGA/CD NPs was 10 times higher than that of Taxol®, and 2.5 times higher than that of PTX PLGA NPs. The AUC of PTX PLGA/CD NPs was 2.6 times that of Taxol® and 2.3 times that of PTX PLGA NPs.

Surprisingly, PTX PLGA/CD NPs displayed raised t1/2, MRT and AUC compared to Taxol^®^, which may indicate rapid tissue distribution of PTX PLGA/CD NPs. Moreover, due to slow PTX release and the high binding constant, the concentration peak of PTX could coincide with a burst release and lead to a prolonged long-term PTX release (Section 3.3) in tissues, since only 80% of PTX was released after 80 h. Probably, the high association of PTX with serum albumin was responsible for the long blood circulation 34.

Abbreviations:

t1/2α(h), distribution phase half-life (T1/2α), applicable to the pharmacokinetic model of two compartments and above, indicating the half-life of the comprehensive concentration change during the elimination of the drug from the central compartment and the distribution of the peripheral compartment;

t1/2β(h), elimination phase half-life (T1/2β), it applies to the pharmacokinetic model of two-compartment and above, which means that after the distribution of the drug between the central compartment and the peripheral compartment reaches a balance, the macroscopically only reflects the half-life of the concentration change eliminated from the central compartment;

MRT, the mean residence time (MRT), another measurement of the elimination process, refers to the average time that the drug molecule stays in the body after rapid intravenous injection;

The plasma concentration curve encloses the area under the drug-time curve (AUC) against the time axis. This parameter is an essential indicator for evaluating the degree of drug absorption, reflecting the exposure characteristics of the drug in the body;

CL, clearance (CL) is the apparent volume distribution of the drug removed from the body per unit time.

The in vivo pharmacokinetic results indicated that PLGA/CD nanoparticle carrier could delay the elimination of paclitaxel in vivo, prolong the retention time of paclitaxel drug in vivo, and improve the bioavailability of paclitaxel in vivo.

### Body accumulation

*In vivo* imaging experiments in mice can visualize the effect of nanoformulation accumulation *in vivo*. After intravenous injection into the blood, the nano-drugs are distributed to various organs and tissues with different affinity to different organs, showing different accumulation effects 35.

The tracer nanoparticles (RDM PLGA NPs, RDM PLGA/CD NPs) containing rhodamine (RDM) were prepared by replacing paclitaxel with rhodamine according to the preparation method of paclitaxel PLGA nanoparticles, and their particle size and potential were measured. the average particle size of RDM PLGA NPs was 160.86nm and ζ-potential was -20.63mV, and the average particle size of RDM PLGA NPs was 154.4nm and ζ-potential was -13.10mV compared with PTX PLGA NPs and PTX PLGA/CD NPs. The average particle size of RDM PLGA/CD NPs was 154.4nm and the ζ-potential was -13.10mV, and the difference in particle size charge was not significant compared with PTX PLGA NPs and PTX PLGA/CD NPs, which could be used for subsequent experiments.

**Figure 4:**
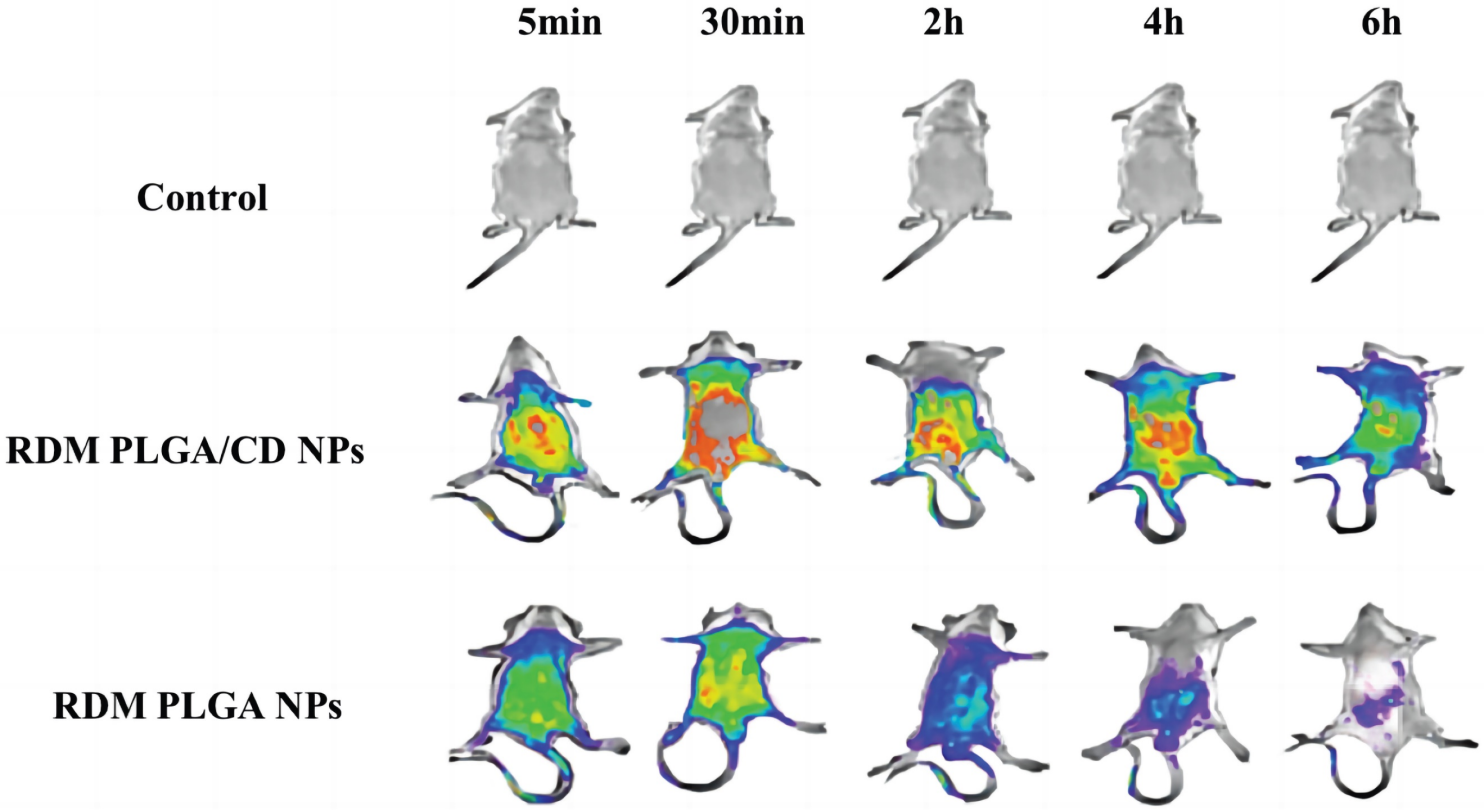
Real-time fluorescence distribution in mice.

The real-time dynamic distribution of the missing nanoparticles in mice is shown in Figure 4, with the fluorescence intensity showing a change from violet, blue, green, yellow to red from low to high. Compared with RDM PLGA NPs, the CD modification can significantly promote the retention of nanoparticles *in vivo*, and the strong fluorescence of RDM PLGA/CD NPs can still be observed *in vivo* at 6h. And the fluorescence signal of CD modified nanoparticles group was higher than that of PLGA NPs group at 5min, 30min, 2h, 4h and 6h. The fluorescence signal of RDM PLGA NPs *in vivo* decreased significantly after 2h. The fluorescence signal of RDM PLGA NPs *in vivo* was significantly lower than that of PLGA NPs. Judging from the site of fluorescence emission, the fluorescence signal of RDM-NPs indicated enrichment in liver/kidney after 30min, and basically concentrated in kidney and bladder excretion after 4h. CD-modified RDM nanoparticles basically concentrated in liver, spleen and lung after 30min, and fluorescence appeared in kidney and bladder after 4h. Judging from the sites of fluorescence emission, the fluorescence was mainly distributed in the liver, lung and spleen in the early stage, and the fluorescence was mainly distributed in the kidney and bladder after 4h, presumably excreted by the kidney.

**Figure 5:**
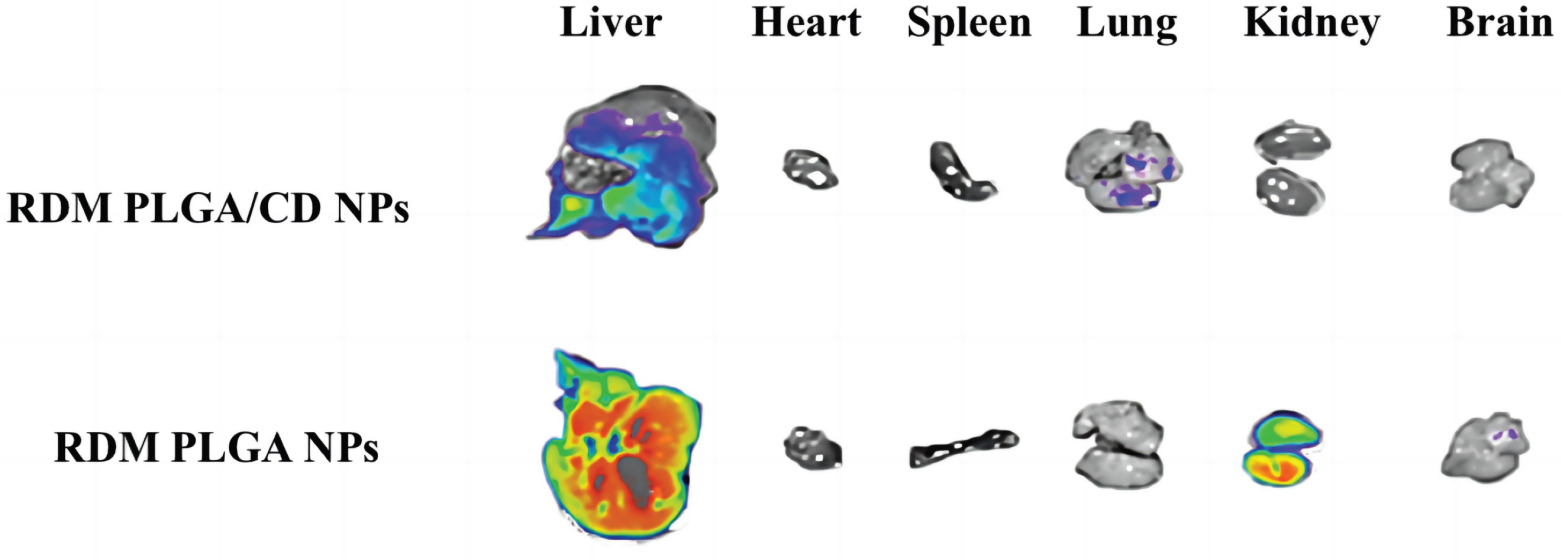
Fluorescence distribution in isolated organs.

The imaging of isolated organs of mice is shown in Figure 5, and it can be seen that after 6h, the unmodified nanoparticles are mainly enriched in the liver, which may be due to the immune recognition of nanoparticles by the reticuloendothelial system, captured and phagocytosed by liver Kuffer cells, and passively targeted to liver tissue through normal physiological processes 36, 37.

The fluorescence signal of the modified nanoparticles was observed in the lung, and the fluorescence signal of the liver tissue was relatively weak, probably due to the fact that a significant portion of the nanoparticles were modified by hydrophilic surface to achieve a "cloaking" effect, avoiding the clearance effect of the immune system and prolonging the circulation time *in vivo* 38.

In general, nanoparticles with small particle size and hydrophilic surface modification are able to avoid phagocytosis by the reticuloendothelial system (RES) and promote long *in vivo* circulation. Larger particle size and surface unmodified hydrophilic nanoparticles have more extensive binding of conditioners and consequently enhance phagocytosis and uptake into the liver, leading to hepatic accumulation 21.

The effect of CD modification on the *in vivo* circulation time of nanosystems can be clearly presented in a live animal model, where CD-modified nanoparticles exhibit enhanced *in vivo* retention and prolonged *in vivo* circulation time, and the present experiments provide a non-invasive method for *in vivo* distribution studies of nanoparticles.

## Conclusions

Well-defined CD-modified PLGA nanoparticles was synthesized through the emulsification solvent evaporation method. It was then used as the building unit for the development of NPs, including CD at the core/shell intermediate layer. We found that the PTX release pattern from CD-modified PLGA NPs was biphasic. PTX-loaded NPs were characterized by a sharp burst release phase. Similar results were described for PLGA nanoparticles containing lipophilic drugs 39. It was shown that PTX encapsulation demonstrated an absence of hemolytic activity and potential for application in nanomedicine. The results of the *in vivo* distribution and pharmacokinetics study showed that the prepared nanoparticles had a longer retention time and circulation time within the body. In addition, nano-drugs have higher aggregation in lungs and kidney tissues. This study indicate that CD can significantly improve the pharmacokinetic behaviour of PTX-loaded PLGA nanoparticles. This effect may be attributable to the action of 2-HP-β-CD improving tumor aggregation and permeability. The problem of passage of the nano-drug through the capillary wall to reach the tumor tissue's surface and reaching the tumor's deep layer through the complex microenvironment matrix under high interstitial pressure still needs to be addressed in further studies. We can conclude that the combination of CD and PLGA nanoparticles can increase the penetration of the drugs, and thus, their concentration in the tumor.

## Figures and Tables

**Table 1:** Nanoparticle characterization results(n=3)

Preparation	Size(nm)	PDI	ζ(mV)	D90(nm)	EE(%)
PTX PLGA NPs	163.57±2.07	0.28±0.08	-(20.53±2.79)	284.20±10.64	60.44±6.80
PTX PLGA/CD NPs NNNPs	148.57±1.66	0.14±0.01	-(11.42±0.84)	256.10±4.65	85.70±2.06

**Table 2:** Pharmacokinetic parameters of NPs and Taxol® (Compared to Taxol®, ***P<0.001; n=6)

	Taxol^®^	PTX PLGA NPs	PTX PLGA/CD NPs
t_1/2α_(h)	0.32±0.10	0.20±0.03	0.27±0.02
t_1/2β_(h)	1.43±1.34	2.75±1.43	9.23±3.58^***^
MRT_0-t_(h)	1.74±0.61	1.14±0.42	2.31±0.18
MRT_0-∞_(h)	2.29±0.46	9.42±14.01	23.48±25.19^***^
AUC_0-t_(ng·L^-1^·h)	5539.36±1859.73	6030.09±541.22	10152.88±1470.54^***^
AUC_0-∞_(ng·L^-1^·h)	6383.63±3022.91	7092.40±1520.21	16466.73±5189.89^***^
C_max_(ng·L^-1^)	5828.35±170.70	10639.20±408.68^***^	12494.06±2664.56^***^
CL(L·h^-1^·kg)	4.31±0.08	2.90±0.53	1.30±0.33

## Abbreviations

t1/2α(h): distribution phase half-life (T1/2α)
t1/2β(h): elimination phase half-life (T1/2β)
MRT: the mean residence time (MRT)
AUC: area under curve
CL: clearance
